# Protocol for a systematic review and meta-analysis of hepatitis C virus (HCV) prevalence and incidence in the Horn of Africa sub-region of the Middle East and North Africa

**DOI:** 10.1186/2046-4053-3-146

**Published:** 2014-12-16

**Authors:** Karima Chaabna, Yousra A Mohamoud, Hiam Chemaitelly, Ghina R Mumtaz, Laith J Abu-Raddad

**Affiliations:** Infectious Disease Epidemiology Group, Weill Cornell Medical College—Qatar, Cornell University, Qatar Foundation—Education City, Doha, Qatar; Department of Healthcare Policy and Research, Weill Cornell Medical College, Cornell University, New York, NY USA

**Keywords:** Hepatitis C, Incidence, Prevalence, Epidemiology, Middle East and North Africa, Djibouti, Somalia, Sudan, Yemen

## Abstract

**Background:**

In the Middle East and North Africa (MENA), hepatitis C virus (HCV) distribution appears to present a wide range of prevalence. The scale and nature of HCV disease burden is poorly known in the Horn of Africa sub-region of MENA including Djibouti, Somalia, and Sudan in addition to Yemen at the southwest corner of the Arabian Peninsula. The aim of this review is to provide a systematic review and synthesis of all epidemiological data on HCV prevalence and incidence among the different population groups in this sub-region of MENA. A second aim of the study is to estimate the national population-level HCV prevalence for each of these four countries.

**Methods/design:**

The systematic review will be conducted based on the items outlined in the PRISMA statement. PubMed, Embase, and the World Health organization (WHO) regional databases will be searched for eligible studies without language or date restrictions. Observational and intervention studies reporting data on the prevalence or incidence of HCV in any population group in Djibouti, Somalia, Sudan, or Yemen will be included. Additional sources will be obtained through the database of the MENA HIV/AIDS Epidemiology Synthesis Project, including international organizations’ reports and country-level reports, and abstracts of international conferences. Study and population characteristics will be extracted from eligible publications, with previously agreed *pro formas*; and entered into a computerized database. We will pool prevalence using DerSimonian and Laird random-effects models after a Freeman-Tukey transformation to stabilize variances. We will conduct meta-regression analysis to explore the effect of study-level characteristics as potential sources of heterogeneity.

**Discussion:**

This proposed systematic review and meta-analysis aims to better describe HCV infection distribution across countries in the Horn of Africa sub-region of MENA; and between sub-population groups within each country. The study will provide empirical evidence necessary for researchers, policy-makers, and public health stakeholders to set research, policy, and programming priorities for HCV prevention, control, and treatment.

**Systematic review registration:**

PROSPERO CRD42014010318

**Electronic supplementary material:**

The online version of this article (doi:10.1186/2046-4053-3-146) contains supplementary material, which is available to authorized users.

## Background

Despite recent progress in hepatitis C virus (HCV) drug-related research [1, 2], HCV remains a serious public health issue, with no vaccination yet available [3]. The estimated prevalence of HCV infection worldwide is 2.8% [4]. Region-specific estimates range from <1.0% in northern Europe [5] to >3.0% in North Africa [4, 6]. The largest population-level prevalence of HCV infection in the world is found in Egypt with 14.7% of the adult population being exposed to the infection [7, 8]. Iatrogenic HCV transmission through unsafe (therapeutic) injections, among other parenteral exposures, has sustained transmission of the infection in different countries in earlier decades [9, 10] and appears to persist in some of the low-income countries [11, 12].

The aim of this study is to provide a systematic review and synthesis of all epidemiological data on HCV prevalence and incidence among the different population groups in the Horn of Africa sub-region of the Middle East and North Africa (MENA) including Djibouti, Somalia, and Sudan in addition to Yemen at the southwest corner of the Arabian Peninsula. A second aim of the study is to estimate the national population-level HCV prevalence for each of these four countries. The combined population of this sub-region is approximately 85 million, or over one-fifth of the population of MENA [13]. This MENA sub-region shares socio-cultural and socio-economic similarities and geographic proximity that warrant covering it within the context of one study.

The proposed review is part of a larger project, the MENA HCV Synthesis Project, an ongoing effort to characterize HCV epidemiology in all 23 countries of the MENA region. The ultimate goal of this project is to provide the empirical evidence necessary for researchers, policy-makers, and public health stakeholders to set research, policy, and programming priorities for HCV prevention, control, and treatment in the MENA region.

## Methods

### Data sources and search strategy

The review will be conducted based on the items outlined in the PRISMA statement [14]. PubMed, Embase, and the World Health Organization (WHO) regional databases (WHO African Index Medicus [15] and WHO Index Medicus for the Eastern Mediterranean Region [16]) will be searched for eligible studies with no language or date restrictions. MeSH, Emtree, and free-text terms grouped in two categories will be used: hepatitis C and location (Africa and Yemen). The Boolean operators “AND” will combine the categories and “OR” will join the terms within each category, respectively. Search terms and keywords will be altered as per specification of individual databases. To ensure that we will identify all relevant articles, the search for the African countries (Djibouti, Somalia, and Sudan) will cover the whole African continent, and we will select manually the relevant articles. Yemen is technically in Asia and therefore will be included explicitly in the search terms. In addition, the search will incorporate spelling and acronym variants (for example: VHC, HVC, hépatite) and nationality adjective (for example: African and Yemeni). Specific search criteria are provided in Additional file 1.

The database searches will be supplemented by searching through citations and references of all included papers and identified reviews. Additional sources will be obtained through the database of the MENA HIV/AIDS Epidemiology Synthesis Project [17, 18], including international organizations’ reports and country-level reports, and abstracts of international conferences, and by contacting authors.

### Inclusion and exclusion criteria

Studies will be considered eligible for inclusion in the proposed review if they are performed in one of the four countries of interest: Djibouti, Somalia, Sudan, and Yemen. Eligible studies must include data on the number or frequency (sero-prevalence and/or sero-incidence) of individuals exposed to HCV in any given population at a specific time or for a given period of time. Only studies referring explicitly to HCV and published after the discovery of HCV in 1989 will be considered for the review. Studies referring to HCV as non-A non-B hepatitis will be excluded.

We will include observational and intervention studies with primary data using cross-sectional, case–control, and cohort (prospective and retrospective) designs. For case–control studies of different conditions, we will extract HCV prevalence among cases and controls separately if available. For cohort studies, we will extract HCV incidence and/or HCV prevalence (but only at baseline for prevalence). For intervention studies, we will extract HCV incidence and/or HCV prevalence (but only at baseline for prevalence) for the intervention and control arms separately if available. Case reports, case series, reviews, qualitative studies, editorials, commentaries, letters to editors, author replies, and animal studies will be excluded. In addition, any study with fewer than 15 participants will not be included.

Studies may be conducted in a variety of settings (such as clinics, hospitals, households, communities, etc.) and in any population group. Both sexes and all age groups from any racial or ethnic population will be included. Exposure to HCV infection must be ascertained using a clearly specified laboratory test. Studies using self-report to identify HCV status will be excluded from the review.

### Screening strategy

All citations obtained using the search strategy will be imported into Endnote (Endnote X7, Thomson Reuters, San Francisco, CA), and duplicates will be deleted. The remaining unique and potentially relevant records will be imported into Microsoft Excel where screening for relevance and eligibility will take place. The titles and abstracts of all retrieved records will be screened for relevance by Karima Chaabna (KC). Full text of all articles deemed relevant after the initial title and abstract screening will be retrieved and assessed for eligibility by KC. Any remaining non-eligible articles will be excluded. Reasons for exclusion will be recorded.

### Data extraction

Relevant data will be extracted by one reviewer (KC) from eligible publications into a predefined extraction Excel sheet developed for this review. All extracted data will be checked by a second reviewer (Silva Kouyoumjian; SK) to assess and ensure the quality of extraction. The checking will consist of verifying whether, for each study, all extracted variables are correctly included in the database. Inconsistencies between reviewers will be discussed among the study team and sorted out by consensus.

The following data will be extracted from each eligible study included in the review: author(s), year of publication, title, journal, study location, year(s) of data collection, study design, sampling technique, population profile (such as blood donors, barbers, health care workers, people who inject drugs), and socio-demographic characteristics of the population (sex and age). Furthermore, we will extract the name of the serological test used to determine HCV exposure, the number of participants included and that did participate, the response rate, and the raw results obtained for the primary outcome.

Although not part of the inclusion criteria, the following data will be extracted from included studies when available: HCV RNA prevalence and incidence, HCV genotype frequency measures among HCV antibody positive patients, and risk factors for HCV infection with significant unadjusted and adjusted odds ratio (*p*-value ≤ 0.05).

Relevant data will be extracted from abstracts for studies whose full text could not be obtained even after contacting the authors. Data from any article providing multiple HCV measures for different population subgroups, sex, and/or several study periods will be extracted as several studies and treated as separate datasets in the analysis.

### Risk of bias and quality assessment

We will incorporate risk of bias assessment into our analyses by evaluating sources of bias that may affect the overall estimations. The methodology for the quality assessment will be adapted from that used recently by two of the co-authors in a systematic review and data synthesis of HIV prevalence and incidence among people who inject drugs in MENA [19]. Based on the Cochrane approach [20], each HCV prevalence measure extracted will be classified as having a low, high, or unknown risk of bias in three domains: sampling methodology, HCV ascertainment, and response rate. Low risk of bias in these domains will be respectively defined as 1) probability-based sampling, 2) biological assays, and 3) response rate ≥80% of the target sample size.

A minimum simple size will be calculated to differentiate estimates with good precision. The minimum sample size will be calculated using the pooled estimate of HCV prevalence among the general population in the included countries [21]. This calculation will utilize the exact binomial (Clopper-Pearson) confidence interval formula [21]. Studies classified as “with good precision” should have a sample size equal to or higher than this calculated minimum sample size. Small-study effect on the effect size will be explored by the use of funnel plots [22], and asymmetry will be tested using Egger’s test [23]. Sensitivity analysis restricted to studies at low risk of bias will be performed.

### Ethics statement

Our review will not require an ethics committee approval or written informed consent because it relies entirely on published data.

### Data synthesis

We will calculate 95% confidence intervals for all HCV prevalence measures with the Clopper-Pearson method [21, 24]. The data will be synthesized by country and by study population for HCV exposure. Four population groups are defined for this study: (1) high risk groups (such as hemodialysis patients and people who inject drugs); (2) intermediate risk groups (such as healthcare workers, prisoners, barbers, and children and spouses of index patients); (3) general population groups which represent the low risk groups (such as blood donors, pregnant women, and healthy populations); and (4) special clinical groups (such as patients with non-Hodgkin lymphoma (NHL), hepatocellular carcinoma (HCC), and chronic and acute viral hepatitis, among other populations where the risk of exposure to HCV infection is uncertain).

### Data analysis

In the framework of the current systematic review, we plan to estimate pooled HCV prevalence and incidence by population subgroup (high risk population, intermediate risk population general population, and special clinical population) in each country included in our analysis. Inverse-variance weighted random-effects models will be used following the conventional method of DerSimonian and Laird [25] after stabilizing the variance by transforming the proportions using the Freeman-Tukey double-arcsine method [26]. The back-transformed pooled proportions will be calculated using Miller’s inverse transformation with the harmonic mean of the sample sizes [27]. The value 0.5 will be added to all cell frequencies of studies with a zero cell count [28]. Forest plots will be generated displaying prevalence (or incidence) with the corresponding 95% confidence intervals for each study and the overall random-effects pooled estimate with its confidence interval [29].

To identify heterogeneity between studies, we will visually inspect the Forest plots, perform Cochran’s Q test [30], and estimate the between-study variance of the true effect sizes (τ^2^) using the DerSimonian and Laird method [25, 31]. To examine the magnitude of the variation between studies due to heterogeneity rather than chance, and whether it impacts the conclusions of the meta-analyses, we will quantify the heterogeneity by using the I^2^ measure and its confidence interval [32]. We will consider a two-sided probability value <0.10 as significant.

A meta-regression will be conducted to identify sources of between-study heterogeneity in the pooled prevalence (or incidence) estimates [33]. Several potential sources of heterogeneity will be specified a priori. The factors considered will be those related to the characteristics of studies (such as risk of bias and year of publication); or sub-populations (such as blood donors and pregnant women). A multivariate meta-regression model will be built by adding each variable sequentially starting with the variable that shows the strongest association with HCV prevalence (or incidence) in a univariate analysis. A variable will remain in the multivariate model if it will be independently associated with HCV prevalence at *p* ≤ 0.10.

Statistical analyses will be performed with Stata v.13.1 (StataCorp, College Station, TX, USA) and R v. 3.1.1.

## Discussion

This systematic review and meta-analysis will define the distribution of HCV prevalence and incidence across countries in the Horn of Africa sub-region of MENA including Djibouti, Somalia, Sudan, and Yemen; and between sub-population groups within each country. The rigorous systematic review and meta-analysis methodology used in this study will ensure a robust knowledge synthesis of available data. The study will provide empirical evidence necessary for researchers, policy-makers and public health stakeholders to set research, policy and programming priorities for HCV prevention, control, and treatment in MENA.

## Electronic supplementary material

Additional file 1: **Search criteria.** Search terms for PubMed and Embase electronic databases. (DOCX 17 KB)

## Abbreviations

CI: confidence interval
HCC: hepatocellular carcinoma
HCV: hepatitis C virus
MENA: Middle East and North Africa
NHL: non-Hodgkin lymphoma
PRISMA: preferred reporting items for systematic reviews and meta-analyses
*p*-value: probability value
WHO: World Health Organization.
