# Supplementary material for: Protocol for a systematic review and meta-analysis of hepatitis C virus (HCV) prevalence and incidence in the Horn of Africa sub-region of the Middle East and North Africa
Source: Syst Rev. 2014 Dec 16;3:146. doi: 10.1186/2046-4053-3-146 (PMC4274704; doi:10.1186/2046-4053-3-146)
Supplement: Supplementary file 1 — Additional file 1: Search criteria. Search terms for PubMed and Embase electronic databases. (DOCX 17 KB) [file 13643_2014_311_MOESM1_ESM.docx]

**Protocol for a systematic review and meta-analysis of hepatitis C virus (HCV) prevalence and incidence in the Horn of Africa sub-region of the Middle East and North Africa**

**Additional file 1**

**Search Criteria**

**PubMed**

("Hepatitis C"[Mesh] OR "Hepatitis C Antibodies"[Mesh] OR "Hepatitis C Antigens"[Mesh] OR "Hepacivirus"[Mesh] OR "Hepatitis C, chronic/epidemiology"[Mesh] OR "Hepatitis C, chronic/etiology"[Mesh] OR "Hepatitis C, chronic/transmission"[Mesh] OR "Hepatitis C, chronic/virology"[Mesh] OR "Hepatitis C"[Text] OR "HCV"[Text] OR "Hepatite"[Text] OR "VHC"[Text] OR "HVC"[Text]) AND ("Yemen"[Mesh] OR "Djibouti"[Mesh] OR "Somalia"[Mesh] OR "Sudan"[Mesh] OR "Africa"[Mesh] OR "Yemen"[Text] OR “Yemeni” [Text] OR "Djibouti"[Text] OR "Somalia"[Text] OR “Somali” [Text] OR "Sudan"[Text] OR “Sudanese” [Text] OR " Africa"[Text] OR “African” [Text])

**Embase**

(Yemen*.mp. or exp Yemen/ or Djibouti.mp. or exp Djibouti/ or Somali*.mp. or exp Somalia/ or Sudan*.mp. or exp Sudan/ or Africa*.mp. or exp Africa/) AND (exp hepatitis C/ or exp Hepatitis C virus/ or hepatitis C.mp. or HCV.mp. or hepacivirus.mp. or Hepatite.mp. or VHC.mp. or HVC.mp.)
